# Factors Associated With Treatment Failure in Moderately Severe Community-Acquired Pneumonia: A Secondary Analysis of a Randomized Clinical Trial

**DOI:** 10.1001/jamanetworkopen.2021.29566

**Published:** 2021-10-15

**Authors:** Aurélien Dinh, Clara Duran, Jacques Ropers, Frédérique Bouchand, Benjamin Davido, Laurène Deconinck, Morgan Matt, Olivia Senard, Aurore Lagrange, Guillaume Mellon, Ruxandra Calin, Sabrina Makhloufi, Victoire de Lastours, Emmanuel Mathieu, Jean-Emmanuel Kahn, Elisabeth Rouveix, Julie Grenet, Jennifer Dumoulin, Thierry Chinet, Marion Pépin, Véronique Delcey, Sylvain Diamantis, Daniel Benhamou, Virginie Vitrat, Marie-Christine Dombret, Didier Guillemot, Bertrand Renaud, Yann-Erick Claessens, José Labarère, Philippe Aegerter, Jean-Pierre Bedos, Anne-Claude Crémieux

**Affiliations:** 1 Infectious Diseases Unit, Raymond-Poincaré University Hospital, Assistance Publique–Hôpitaux de Paris (APHP) Paris Saclay University, Garches, France; 2 Epidemiology and Modeling of Bacterial Evasion to Antibacterials Unit, Institut Pasteur, Paris, France; 3 Clinical Research Unit, Pitié-Salpétrière University Hospital, APHP, Paris, France; 4 Pharmacy Department, Raymond-Poincaré University Hospital, APHP Paris Saclay, Garches, France; 5 Infectious Disease Department, Bichat University Hospital, APHP, University of Paris, Paris, France; 6 Infectious Disease Department, Marne La Vallée Hospital, Grand Hôpital de l'Est Francilien, Marne La Vallée, France; 7 Pneumology Department, Pontoise Hospital, Pontoise, France; 8 Internal Medicine, Beaujon University Hospital, APHP, Clichy, France; 9 Emergency Medicine, Foch Hospital, Suresnes, France; 10 Internal Medicine, Ambroise-Paré University Hospital, APHP Paris Saclay, Boulogne-Billancourt, France; 11 Emergency Medicine, Ambroise-Paré University Hospital, APHP Paris Saclay, Boulogne-Billancourt, France; 12 Pneumology Department, Ambroise-Paré University Hospital, APHP Paris Saclay, Boulogne-Billancourt, France; 13 Geriatric Department, Ambroise-Paré University Hospital, APHP Paris Saclay, Boulogne-Billancourt, France; 14 Internal Medicine, Lariboisière University Hospital, APHP, Paris, France; 15 Infectious Disease Department, Melun Hospital, Melun, France; 16 Pneumology Department, Rouen University Hospital, Rouen, France; 17 Infectious Disease, Annecy Hospital, Annecy, France; 18 Pneumology Department, Bichat University Hospital, APHP, Paris, France.; 19 Emergency Department, Cochin University Hospital, APHP, Paris, France; 20 Emergency Department, Princesse Grace University Hospital, Monaco, France; 21 Quality of Care Unit, Grenoble University Hospital, Grenoble Alpes University, Grenoble, France; 22 UMRS 1168 VIMA, INSERM, Versailles Saint-Quentin University, Versailles, France; 23 Intensive Care Unit, Le Chesnay Hospital, Versailles, France; 24 Infectious Diseases Unit, Saint-Louis University Hospital, APHP, Paris, France

## Abstract

**Importance:**

Failure of treatment is the most serious complication in community-acquired pneumonia (CAP).

**Objective:**

To assess the potential risk factors for treatment failure in clinically stable patients with CAP.

**Design, Setting, and Participants:**

This secondary analysis assesses data from a randomized clinical trial on CAP (Pneumonia Short Treatment [PTC] trial) conducted from December 19, 2013, to February 1, 2018. Data analysis was performed from July 18, 2019, to February 15, 2020. Patients hospitalized at 1 of 16 centers in France for moderately severe CAP who were clinically stable at day 3 of antibiotic treatment were included in the PTC trial and analyzed in the per-protocol trial population.

**Interventions:**

Patients were randomly assigned (1:1) on day 3 of antibiotic treatment to receive β-lactam (amoxicillin-clavulanate [1 g/125 mg] 3 times daily) or placebo for 5 extra days.

**Main Outcomes and Measures:**

The main outcome was failure at 15 days after first antibiotic intake, defined as a temperature greater than 37.9 °C and/or absence of resolution or improvement of respiratory symptoms and/or additional antibiotic treatment for any cause. The association among demographic characteristics, baseline clinical and biological variables available (ie, at the first day of β-lactam treatment), and treatment failure at day 15 among the per-protocol trial population was assessed by univariate and multivariable logistic regressions.

**Results:**

Overall, 310 patients were included in the study; this secondary analysis comprised 291 patients (174 [59.8%] male; mean [SD] age, 69.6 [18.5] years). The failure rate was 26.8%. Male sex (odds ratio [OR], 1.74; 95% CI, 1.01-3.07), age per year (OR, 1.03; 95% CI, 1.01-1.05), Pneumonia Severe Index score (OR, 1.01; 95% CI, 1.00-1.02), the presence of chronic lung disease (OR, 1.85; 95% CI, 1.03-3.30), and creatinine clearance (OR, 0.99; 95% CI, 0.98-1.00) were significantly associated with failure in the univariate analysis. When the Pneumonia Severe Index score was excluded to avoid collinearity with age and sex in the regression model, only male sex (OR, 1.92; 95% CI, 1.08-3.49) and age (OR, 1.02; 95% CI, 1.00-1.05) were associated with failure in the multivariable analysis.

**Conclusions and Relevance:**

In this secondary analysis of a randomized clinical trial, among patients with CAP who reached clinical stability after 3 days of antibiotic treatment, only male sex and age were associated with higher risk of failure, independent of antibiotic treatment duration and biomarker levels. Another randomized clinical trial is needed to evaluate the impact of treatment duration in populations at higher risk for treatment failure.

## Key Points

**Question:**

What are the risk factors for treatment failure in patients with community-acquired pneumonia (CAP) who reached clinical stability after 3 days of β-lactam treatment?

**Findings:**

In this secondary analysis of a randomized clinical trial that included 291 adults, only male sex and age were associated with failure in the multivariable analysis. These results were independent of antibiotic treatment duration and biomarker levels.

**Meaning:**

In this study, among patients with CAP who reached clinical stability after 3 days of antibiotic treatment, male sex and age were associated with higher risk of failure, suggesting that these factors should be taken in account in the treatment of patients with the condition.

## Teaser

This secondary analysis of a randomized clinical trial assesses treatment failure risk factors in patients with community-acquired pneumonia who reached clinical stability after 3 days of β-lactam treatment.

## Introduction

Up to 5.6 million cases of community-acquired pneumonia (CAP) occur annually in the US,^1^ resulting in 600 000 to 800 000 hospitalizations, with the highest incidence rate in older patients.^1,2,3,4^ Community-acquired pneumonia is a heterogeneous disease that ranges from a mild, self-limiting disease to a severe infection that causes respiratory failure, shock, and death.^5,6^ Treatment failure is the most serious complication. Failure significantly increases the risk of complications, length of hospital stay, and death, especially in patients with severe CAP.^3,7,8,9,10,11,12,13^ The incidence of clinical failure in patients with CAP ranges from 6% to 24%^3,7,8,9,10,11,12^ and can reach up to 31% in patients with severe CAP.^13^ Several risk factors for treatment failure have been identified in the literature, such as age, smoking, malnutrition, previous CAP episodes, and comorbidities (chronic pulmonary disease, asthma, and immunosuppression).^14^ Reaching clinical stability is associated with a high rate of favorable outcomes.^15^ The Pneumonia Short Treatment (PTC) trial was a placebo-controlled randomized clinical trial that studied antibiotic treatment duration (3 vs 8 days of β-lactam treatment) among 310 patients hospitalized with moderately severe CAP who reached clinical stability at day 3 of treatment. In this secondary analysis, we aim to evaluate the risk factors for treatment failure among this specific population.

## Methods

### Study Design, Sites, and Study Population

**Figure 1.**
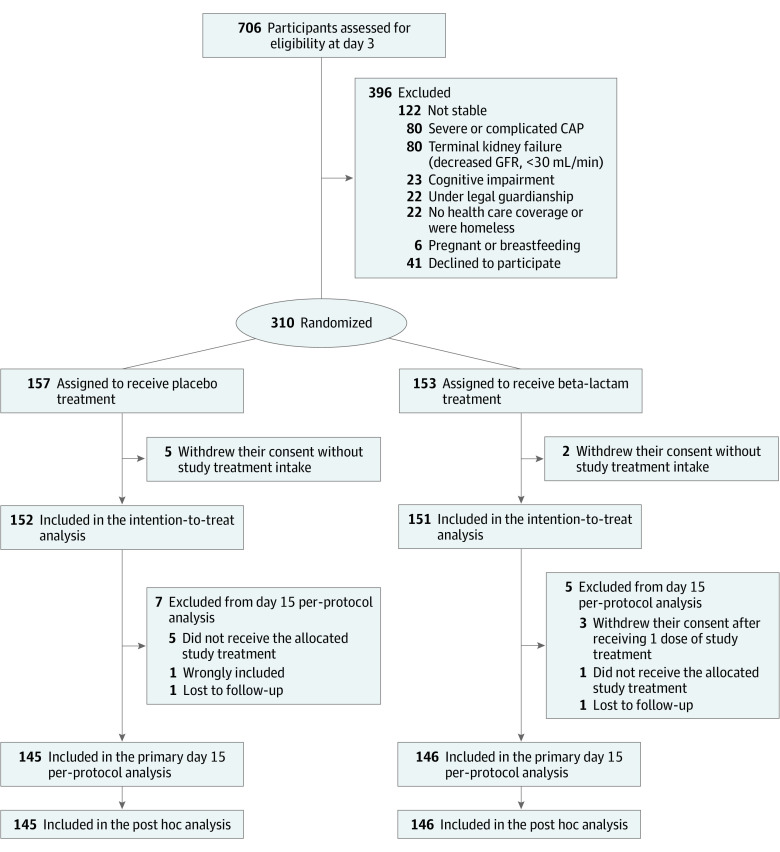
Study Flowchart CAP indicates community-acquired pneumonia; GFR, glomerular filtration rate.

We performed a secondary analysis of a double-blind randomized clinical trial (the PTC trial), which included 310 patients with moderately severe CAP in 16 teaching hospitals in France, from December 19, 2013, to February 1, 2018. Data analysis was performed from July 18, 2019, to February 15, 2020. The flow diagram of the patients in the trial is shown in Figure 1. The primary outcome was treatment failure 15 days after first antibiotic intake, defined as a temperature greater than 37.9 °C and/or absence of resolution or improvement of respiratory symptoms (coughing frequency or severity, sputum production, dyspnea, or crackles) and/or additional antibiotic treatment for any cause. The study design and main results have been published previously.^16^

In brief, patients who met the following inclusion criteria were studied: age of 18 years or older, hospitalized in a non–critical care ward for CAP, treated with β-lactams, chosen at the discretion of the physician in charge to receive amoxicillin-clavulanate (oral or intravenous) or parenteral third-generation cephalosporin (ceftriaxone or cefotaxime), and presented after 72 hours of treatment with a clinical response defined by the presence of stability criteria.^3^ Race and ethnicity data are not allowed to be collected in France for research by law; therefore, these data are not available for analysis. Community-acquired pneumonia was defined as the association of at least 1 acute clinical sign compatible with pneumonia (among dyspnea, cough, purulent sputum, or crackles), temperature greater than 38 °C, and a pulmonary infiltrate on chest radiography. Stability criteria were defined, according to the Infectious Diseases Society of America, as apyrexia (temperature ≤37.8 °C), heart rate less than 100 beats/min, respiratory rate less than 24 breaths/min, arterial oxygen saturation of 90% or higher, systolic blood pressure of 90 mm Hg or higher, and normal mental status.^3^ Main exclusion criteria were signs of severe and/or complicated CAP, known immunosuppression, health care–associated pneumonia or suspicion of aspiration pneumonia, any other infection that necessitated concomitant antibiotic treatment, and/or suspected or confirmed legionellosis. Additional eligibility and exclusion criteria are listed in the eAppendix in Supplement 1.

The trial was approved by the Versailles/Saint-Germain-en-Laye University Ethics Committee, the French National Agency for Medicines and Health Products Safety, and the French Data Protection Agency. The study was performed in accordance with the ethical principles of the Declaration of Helsinki^17^ and the Guidelines for Good Clinical Practice. All participants provided written informed consent, which included the conduct of future studies. All data were deidentified. This study followed the Consolidated Standards of Reporting Trials (CONSORT) reporting guideline.

### Randomization and Masking

After 3 full days (72 hours) of β-lactam treatment, patients with all clinical criteria of stability were randomly assigned in a 1:1 ratio to receive oral amoxicillin-clavulanate (500 mg/62.5 mg) treatment or placebo (2 pills 3 times daily) for 5 extra days. Randomization was performed with stratification according to randomization site and Pneumonia Severity Index (PSI) (score of ≤70 or >70). The PSI is scored from 0 to 395, stratifying patients into classes according to the 30-day risk of death: low risk for classes I and II (scores, 0-70), low risk for class III (scores, 71-90), medium risk for class IV (scores, 91-130), and high risk for class V (scores, 131-395). Patients, treating physicians, investigators, pharmacists, and study coordinators were masked to treatment allocation.

### Variables

We performed this secondary analysis among the per-protocol study population, which included all patients randomly assigned to treatment, patients not erroneously included, patients who received their assigned treatment, and patients who received at least 80% of this treatment, except if discontinuation was attributable to worsening of their condition. Those who withdrew consent after more than 1 dose of study treatment and those lost to follow-up, except if they received additional treatment since day 3, were excluded.

The variables included demographic characteristics, clinical and radiological data, and results of usual blood tests from the first day of β-lactam treatment (day 0). Disease severity within the first 24 hours after diagnosis, determined with the PSI,^18^ antibiotic treatment duration (3 or 8 days), and pneumonia-related symptoms scored using the CAP score^19^ were also included in the secondary analysis (eAppendix in Supplement 1). (The CAP score, which is a clinical score used to quantify subjective CAP symptoms, has been described by Moussaoui et al.^19^ It is a short and reliable questionnaire that evaluates changes in respiratory symptoms and well-being during the treatment of CAP. The CAP score is scored from −5.91 [more severe symptoms] to 101.2 [none or mildest symptoms]; see eTable 1 in Supplement 1.) Visits with the physician in charge of the patients were planned 15 days after the start of antibiotic treatment, and clinical data (stability criteria, CAP score, and adverse events) were recorded.

### Statistical Analysis

Continuous variables are presented as means (SDs) and as tabular descriptions for qualitative characteristics. Normality tests were first performed, and normal (and lognormal) distribution was found for all data. We used χ^2^ tests to compare the distributions of categorical variables, whereas 2-tailed, unpaired *t* tests were used to compare the distributions of quantitative continuous variables. All reported *P* values were based on 2-sided tests, and *P* ≤ .05 was considered statistically significant.

To identify risk factors associated with failure at day 15, a univariate analysis by logistic regression was performed, using demographic and medical characteristics as well as all clinical, biological, and radiological data from day 0 of antibiotic treatment. A multivariable analysis by logistic regression was then performed using all variables from the univariate analysis that had a *P* ≤ .20, except for variables such as PSI score and urea nitrogen level to avoid multicollinearity with other variables from the regression model (eg, age, sex, and creatinine clearance). Odds ratios (ORs) were calculated from the univariate and multivariable analysis to quantify association with failure at day 15 with 95% CIs. An OR greater than 1.00 was considered to be associated with failure. Analyses were performed with the use of R software, version 3.6.1 (R Foundation for Statistical Computing).

## Results

Among the 310 patients included in the PTC trail, the per-protocol analysis at day 15 comprised 291 patients (174 [59.8%] male; mean [SD] age, 69.6 [18.5] years), with a failure rate of 26.8% (n = 78). The characteristics of the included population are presented in Table 1. Main comorbidities were chronic lung disease (68 [23.4%]), heart failure (60 [20.7%]), and diabetes (54 [18.6%]). Main causes of failure were no resolution or improvement of symptoms (62 [79.5%]), additional antibiotic treatment (8 [10.2%]), and fever at day 15 (4 [5.1%]). Only 1 patient in the failure group had died before day 15, after experiencing fever and possible pulmonary edema.

**Table 1.** Characteristics of Study Population at Baseline (First Day of β-Lactam Treatment)^a^

Characteristic	Failure (n = 78)	Cure (n = 213)	*P* value
Sex, No. (%)			
Male	54 (69.2)	120 (56.3)	.05^b^
Female	24 (30.8)	93 (43.7)	
Age, y	76.2 (15.7)	67.2 (18.9)	.01^b^
Comorbidities, No. (%)			
Institutionalized	4 (5.1)	5 (2.4)	.25
Neoplasia	1 (1.3)	5 (2.4)	.55
Liver failure	1 (1.3)	5 (2.4)	.55
Heart failure	18 (23.1)	42 (19.7)	.53
Coronary disease	14 (18.0)	27 (12.7)	.27
Cerebrovascular disease	5 (6.4)	17 (8.0)	.65
Kidney failure	8 (10.3)	15 (7.0)	.38
Diabetes	17 (31.5)	37 (68.5)	.40
Chronic lung disease	25 (32.1)	43 (20.2)	.04^b^
Tobacco use	13 (16.6)	39 (18.3)	.71
Clinical signs at day 0, No. (%)			
Dyspnea	47 (60.3)	114 (53.5)	.31
Cough	65 (83.3)	168 (78.9)	.39
Sputum production	29 (37.2)	80 (37.6)	.95
Crackles	59 (75.6)	164 (77.0)	.81
Confusion	6 (7.7)	19 (8.9)	.73
Pleurisy	2 (2.6)	6 (2.8)	.91
Delay between first symptom and admission, d	5.0 (5.0)	4.6 (5.6)	.61
Patients with 3-day antibiotic treatment, No. (%)	32 (41.0)	114 (53.5)	.06
Antibiotic molecule in the first 3 days, No. (%)			
Amoxicillin-clavulanate	58 (74.3)	130 (61.0)	.06
Third-generation cephalosporins	13 (16.7)	47 (22.1)	.31
Both molecules	7 (9.0)	36 (16.9)	.09
Vital signs at day 0			
Respiratory rate, breaths/min	24.8 (7.5)	24.6 (7.4)	.87
Temperature, °C	38.8 (0.6)	38.9 (0.6)	.48
Arterial pressure, mm Hg			
Systolic	139.5 (24.6)	135.0 (24.0)	.16
Diastolic	75.4 (13.6)	73.4 (15.1)	.30
Heart rate, beats/min	102.2 (20.1)	103.3 (19.5)	.67
Oxygen saturation, %	94.0 (3.7)	94.5 (3.9)	.31
PSI score at day 0	91.3 (29.6)	78.7 (32.2)	.01^b^
Biological parameters at day 0			
Creatinine clearance, mL/min/1.73 m^2^	73.2 (26.1)	80.1 (23.4)	.03^b^
Urea nitrogen, mg/dL	22.7 (11.8)	19.3 (10.4)	.03^b^
Sodium, mEq/L	137.4 (3.7)	137.3 (3.8)	.79
Glucose, mg/dL	122.5 (46.8)	124.3 (46.8)	.78
Hematocrit, %	38.2 (5.1)	38.7 (5.5)	.52
White blood cells, /μL	13 600 (10 900)	12 500 (6000)	.98
Neutrophils, /μL	12 100 (11 900)	10 200 (5300)	.47
Platelets, ×10^3^/μL	248.3 (119.4)	223.1 (89.2)	.06
Procalcitonin, μg/L	1.4 (4.0)	2.6 (7.3)	.35
C-reactive protein, mg/dL	14.3 (11.6)	14.5 (12.6)	.91
Radiography at day 0, No. (%)			
Multilobar infection	17 (21.8)	34 (16.0)	.24
Pleural effusion	8 (10.3)	18 (8.4)	.61
CAP score at day 0	43.8 (19.0)	45.4 (20.7)	.82
Hospital length of stay, d	10.4 (8.7)	7.9 (7.2)	.03^b^

Abbreviations: CAP, community-acquired pneumonia; PSI, Pneumonia Severity Index.

SI conversion: To convert creatinine clearance to milliliters per second per square meters, multiply by 0.0167; urea nitrogen to millimoles per liter, multiply by 0.357; sodium to millimoles per liter, multiply by 1; glucose to millimoles per liter, multiply by 0.0555; hematocrit to a proportion of 1.0, multiply by 0.01; white blood cells and neutrophils to ×10^9^ per liter, multiply by 0.001; platelets to ×10^9^ per liter, multiply by 1; and C-reactive protein to milligrams per liter, multiply by 10.

^a^ Data are presented as mean (SD) unless otherwise indicated.

^b^ Data statistically significant.

The main symptoms present at day 15 among patients with treatment failure were purulent sputum alone (15 [24.2%]), dyspnea alone (14 [22.6%]), cough alone (13 [21.0%]), cough and purulent sputum (11 [17.1%]), cough and crackles (3 [4.8%]), crackles alone (2 [3.2%]), dyspnea associated with purulent sputum (2 [3.2%]), dyspnea associated with cough (2 [3.2%]), dyspnea associated with crackles (1 [1.6%]), cough associated with purulent sputum (1 [1.6%]), and sputum associated with crackles (1 [1.6%]).

**Figure 2.**
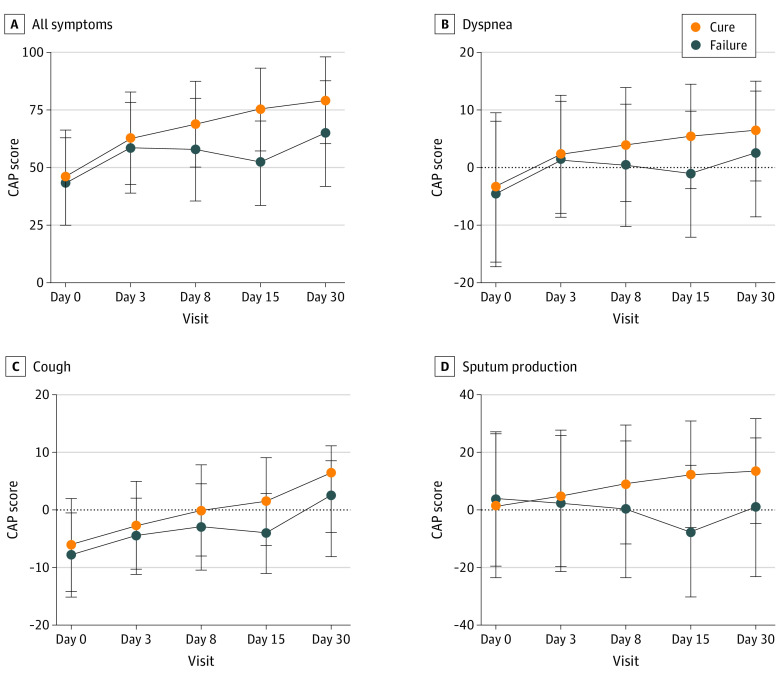
Course of Symptom According to Outcome (Community-Acquired Pneumonia [CAP] Score) Error bars indicate standard deviation. A higher CAP score corresponds to fewer pneumonia symptoms.

Similar evolutions of mean (SD) CAP scores were found in all patients at day 0 (43.8 [19.0] vs 45.4 [20.7], *P* = .61) and at day 3 (58.4 [19.8] vs 62.7 [20.2], *P* = .14). However, patients with treatment failure at day 15 had lower mean (SD) CAP scores at day 8 (57.8 [22.5] vs 69.0 [18.8], *P* < .001) and day 15 (51.9 [18.4] vs 75.3 [18.1], *P* < .001). Furthermore, mean (SD) CAP scores for each respiratory symptom were significantly different between the 2 groups (dyspnea: 0.5 [10.6] vs 4.0 [9.9], *P* = .01 at day 8 and −1.1 [11.1] vs 5.5 [9.1], *P* < .001 at day 15; cough: −2.9 [7.5] vs −0.1 [7.9], *P* = .01 at day 8 and −4.0 [6.9] vs 1.5 [7.6], *P* < .001 at day 15; and sputum production: 0.4 [23.8] vs 8.9 [20.9], *P* = .01 at day 8 and −7.3 [22.9] vs 12.7 [18.6], *P* < .001 at day 15) (Figure 2).

The factors significantly associated with treatment failure at day 15 in univariate analysis (Table 2) were as follows: male sex (OR, 1.74; 95% CI, 1.01-3.07), age per year (OR, 1.03; 95% CI, 1.01-1.05), chronic lung disease (OR, 1.85; 95% CI, 1.03-3.30), PSI score at day 0 (OR, 1.01; 95% CI, 1.00-1.02), creatinine clearance at day 0 (OR, 0.99; 95% CI, 0.98-1.00), and urea nitrogen level at day 0 (OR, 1.07; 95% CI, 1.00-1.14). Systolic arterial pressure at day 0 (OR, 1.00; 95% CI, 1.00-1.02) and platelet count at day 0 (OR, 1.00; 95% CI, 1.00-1.00) had a *P* < .20 and therefore were not statistically significant in the univariate analysis but were included in the multivariable regression model. When PSI score and urea level at day 0 were excluded to avoid multicollinearity, the factors significantly associated with treatment failure at day 15 in multivariable analysis (Table 2) were male sex (OR, 1.92; 95% CI, 1.08-3.49) and age per year (OR, 1.02; 95% CI, 1.00-1.05). Any collinearity between the 2 variables was also verified by comparing the study population’s characteristics according to their sex (eTable 2 in Supplement 1): no significant statistical difference was found between male sex and age (mean (SD) age, 69.4 [18.3] vs 70.0 [18.9] years; *P* = .76).

**Table 2.** Univariate and Multivariate Analysis of Variables Associated With Failure at Day 15

Variable	Univariate analysis		Multivariate analysis	
	OR (95% CI)	*P* value	OR (95% CI)	*P* value
Male	1.74 (1.01-3.07)	.05	1.92 (1.08-3.49)	.03^a^
Age	1.03 (1.01-1.05)	<.001	1.02 (1.00-1.05)	.03^a^
Chronic lung disease	1.85 (1.03-3.30)	.04	1.39 (0.74-2.56)	.30
Creatinine clearance at day 0	0.99 (0.98-1.00)	.03	0.99 (0.98-1.01)	.27
Systolic arterial pressure at day 0	1.01 (1.00-1.02)	.16	1.00 (0.99-1.02)	.56
Platelet count at day 0	1.00 (1.00-1.00)	.06	1.00 (1.00-1.01)	.23

Abbreviation: OR, odds ratio.

^a^ Data are statistically significant.

## Discussion

In this secondary analysis of a randomized clinical trial of patients with CAP who reached clinical stability after 3 days of antibiotic treatment, only male sex and age were associated with higher risk of failure. Our study population is similar to the usual population described in the literature concerning patients hospitalized with CAP, with the exception of patients with immunosuppression, who were excluded from our trial. Indeed, the median age of the population with CAP usually described is between 70 and 74 years, with most being male and having a high number of comorbidities, such as diabetes, chronic obstructive pulmonary disease, chronic heart failure, and a PSI score of III to IV.^20,21,22^

Although approximately half of patients with moderately severe CAP reach clinical stability within 3 days,^15,23^ which is associated with a shorter hospital stay and a better prognosis,^22^ the originality of our study is to focus on those patients and observe their clinical outcome. Our study found that risks factors for treatment failure among patients hospitalized for CAP who reached clinical stability at day 3, in univariate analysis, were age and sex, which are well-known risk factors associated with failure in the literature and may be the most influential characteristics^24^; PSI score, which has been used to evaluate the lethality rate^18^; chronic lung disease; and kidney failure, which is often associated with failure and therefore could be a marker of severity rather than a proper risk factor. In the multivariable analysis, only male sex and age were significantly associated with treatment failure.

Risks factors for failure have been explored in the literature. Despite heterogeneity of failure definition, age and comorbid conditions have also been associated with a higher risk of failure in previous works.^9,12,25^ In a systematic review of the literature,^14^ 29 studies were selected, with 45% of them focused on individuals 65 years or older to determine risk factors for CAP. Several risk factors were identified: age, smoking, environmental exposures, malnutrition, previous CAP, chronic pulmonary disease, asthma, functional impairment, poor dental health, immunosuppressive therapy, oral corticosteroids, and treatment with gastric acid–suppressive drugs. Some of these factors could be corrected, which would reduce morbidity and mortality among adult patients with CAP, particularly among the older patients. Regarding nonmodifiable criteria, age and chronic pulmonary disease were also identified in our study.

In the current study, only a few of these risk factors were significant, possibly because only patients with CAP who reached clinical stability at day 3 were included. As previously reported, reaching clinical stability is associated with a high rate of favorable outcome.^15^ In our original study, patients treated with 3 or 8 days of β-lactam therapy reached the same cure rate. Thus, the antibiotic treatment durations used had no association with failure in this analysis. However, rates of failure vary in the literature, depending on the different definitions used and time of assessment. Moreover, failure could be attributable to noninfectious causes. For instance, in a prospective, multicenter cohort study^8^ performed in hospitalized patients, treatment failure occurred in 15% of patients, with early failure occurring in 62% and late failure in 38%. The causes were infectious in 40%, noninfectious in 16%, and undetermined in 44%. These findings emphasize the difficulty in defining failure and harmonizing criteria to compare study results because risk factors for failure depend on its definition. Overall, the failure rate ranged from 11% to 16%,^8,10,11^ with a rate of early clinical failure varying from 6% to 9%.^8,9^ The failure rate in our trial seems higher than previously reported, especially among patients who reached early clinical stability probably because of the stringent definition of cure in our trial. Indeed, persistence of clinical symptoms (eg, cough) without worsening or additional antibiotic was considered as failure. In fact, our description of failure indicated that the main causes for classification as failure were the persistence or worsening of clinical symptoms. Few patients were classified as having treatment failure for additional antibiotic treatment or death. In addition, the main persistent or worsened symptoms of patients at day 15 were purulent sputum, cough, and dyspnea but not fever. However, these symptoms are usually reported as persistent among patients with pneumonia without indicating treatment failure.^26^

In our study, all patients had similar evolutions of CAP score from day 0 to day 3. The CAP score at day 0 was not associated with failure. However, patients classified as having treatment failure at day 15 had lower CAP scores at day 8 and day 15 for each respiratory symptom. Therefore, this finding is consistent with the study's definition of failure, which included persistence or worsening of respiratory symptoms.

Finally, following biomarker levels was not associated with failure in our study. Indeed, C-reactive protein and procalcitonin levels, which were collected when available, did not seem informative among patients who were stable at day 3. Another study^27^ also found that neither procalcitonin nor leukocyte count was associated with cure. Procalcitonin is an interesting tool to shorten treatment duration, as demonstrated by 2 meta-analyses.^28,29^ Nevertheless, a recent trial^28^ that compared procalcitonin levels and clinical evolution among patients with suspected lower respiratory tract infection found no association with antibiotic prescriptions. Finally, the recent Infectious Diseases Society of America and American Thoracic Society guidelines state that, despite reducing duration of antibiotic therapy, the use of procalcitonin level monitoring does not lead to overall lower durations of treatment compared with recommended durations.^29^ This finding reinforces the importance of clinical examination on biological data.

### Limitations

This study has limitations. Definition of CAP cases and outcomes varies widely among clinical trials. The CAP diagnostic criteria within randomized clinical trials are heterogeneous, which could hinder the validity of their results.^30^ Furthermore, a wide variety of failure definitions for CAP has been used, depending on trials. The US Food and Drug Administration has suggested a definition supported by clinical response, which is difficult to apply to current practice because of its complexity.^31^ However, our study population included only patients who reached stability, which is associated with a favorable prognosis in the literature. Immunocompromised patients, who were likely to have poor outcomes, were excluded from our trial, which would limit the generalizability of our results.

## Conclusions

In this study, among patients with CAP who met the criteria for stability after 3 days of antibiotic treatment, age and sex but not comorbidities or severity of disease were associated with an increased risk of failure. Surveillance of clinical signs (ie, stability criteria) is of paramount importance. These results should be taken in account for the treatment of patients with CAP.

## Notes

Supplement 1. **eAppendix.** Supplementary Methods **eTable 1.** CAP Score Questionnaire and Calculation Scheme **eTable 2.** Characteristics of Study Population at Baseline (ie, First Day of β-Lactam) According to Sex
eReferences

Supplement 2. **Nonauthor Collaborators.** Pneumonia Short Treatment Study Group Members
