# Supplementary material for: Factors Associated With Treatment Failure in Moderately Severe Community-Acquired Pneumonia: A Secondary Analysis of a Randomized Clinical Trial
Source: JAMA Netw Open. 2021 Oct 15;4(10):e2129566. doi: 10.1001/jamanetworkopen.2021.29566 (PMC8520128; doi:10.1001/jamanetworkopen.2021.29566)
Supplement: Supplement 1. — eAppendix. Supplementary Methods eTable 1. CAP Score Questionnaire and Calculation Scheme eTable 2. Characteristics of Study Population at Baseline (ie, First Day of β-Lactam) According to Sex eReferences [file jamanetwopen-e2129566-s001.pdf]

Supplementary Online Content

Dinh A, Duran C, Ropers J, et al; Pneumonia Short Treatment (PTC) Study Group. Factors associated with treatment failure in moderately severe community-acquired pneumonia: a secondary analysis of a randomized clinical trial. *JAMA Netw Open*. 2021;4(10):e2129566. doi:10.1001/jamanetworkopen.2021.29566

eAppendix. Supplementary Methods

eTable 1. CAP Score Questionnaire and Calculation Scheme

eTable 2. Characteristics of Study Population at Baseline (ie, First Day of β-Lactam) According to Sex

eReferences

This supplementary material has been provided by the authors to give readers additional information about their work.

© 2021 Dinh A et al. *JAMA Network Open.*

eAppendix. Supplementary Methods

1. **Inclusion and exclusion criteria**

   1.1. **Inclusion criteria**

Patients are eligible when:

- aged 18 years of age or older,
- hospitalized in a non-intensive care unit ward with clinically suspected community-acquired pneumonia (CAP) at admission (Day 0), defined as at least 1 acute clinical signs among dyspnea, cough, purulent sputum, crackles,
- having a temperature > 38°C in the 48h prior to admission,
- and a new infiltrate on chest X-ray or CT scan at admission (Day 0) or in the following 3 days (until Day 3),
- who presented an early clinical response after three days of monotherapy with a parenteral third-generation cephalosporin, or oral or intra-venous amoxicillin-clavulanate treatment, defined by the presence at Day 3 of :
    - apyrexia (T°C ≤ 37.8)
    - heart rate < 100/min
    - respiratory rate < 24/min, according to the patient's usual mode of oxygenation,
    - arterial oxygen saturation ≥ 90%, according to the patient's usual mode of oxygenation,
    - systolic blood pressure ≥ 90mmHg,
- able to take an oral antibiotic treatment,
- and giving written informed consent for participating to the study.

© 2021 Dinh A et al. *JAMA Network Open.*

1.2. Exclusion criteria

Patients are not eligible for enrolment in the study when having:

- Signs of severe and/or complicated CAP (abscess, massive pleural effusion, serious chronic respiratory insufficiency)
- Known immunosuppression (asplenia, neutropenia, agammaglobulinemia, transplant, myeloma, lymphoma, known HIV and CD4<400/mm3, sickle-cell disease, Child-Pugh class C cirrhosis)
- Combination therapy for more than 1 dose within the first 3 days
- Suspected or confirmed legionellosis or intra-cellular bacteria
- More than 24h of antibiotics in the 7 days prior to admission
- Terminal renal failure (GFR < 30mL/min)
- Past history of jaundice or liver damage due to amoxicillin/clavulanate
- Allergy or hypersensitivity to β-lactams
- Healthcare-associated pneumonia
- Suspicion of aspiration pneumonia
- Any other infection necessitating concomitant antibiotic treatment
- Pregnancy or breastfeeding
- Life expectancy < 1 month
- Patient under legal guardianship or without healthcare insurance coverage
- Homeless patient

© 2021 Dinh A et al. *JAMA Network Open.*

2. CAP score: presentation

The CAP score (the community-acquired pneumonia score) is a pneumonia-related symptoms score, calculated at days 0, 3, 8, 15 and 30, which enables to evaluate and compare the evolution of CAP symptoms between the two treatment groups [1]. This score is based on a short questionnaire for adults admitted to hospital with mild to moderately severe CAP. It includes 8 items that evaluate CAP symptoms' severity: 1) the presence of dyspnea (graded as presence of dyspnea at rest, while walking around, washing and dressing, going for a walk, showering, or walking-up stairs), and 2) its severity in general (5-point Likert scale); 3) coughing (4-point Likert scale); 4) sputum, 5) coughing up sputum with ease, and 6) color of the sputum, evaluated using a 4-point Likert scale; and 7) the general state of health, and 8) fatigue measured using a visual analogue scale (5 choices). Low values indicate more severe symptoms.

eTable 1. CAP Score Questionnaire and Calculation Scheme

**Items**		**Quantification**
**1.** Are you today bothered by shortness of breath when…		
- sitting still	☐ Yes	1
- walking around the house/ward	☐ Yes	1
- washing/dressing	☐ Yes	1
- walking in the street	☐ Yes	1
- taking a shower	☐ Yes	1
- walking the stairs	☐ Yes	1
Subtotal (sum) :	*0*	6
	1	-2
	2-3	-6
	4-6	-8
**2.** If you were to give a mark on a 1 to 5 scale expressing the severity of your shortness of breath at the moment, which mark would that be?		
☐ not at all short of breath		7
☐ slightly short of breath		-2
☐ fairly short of breath		-8
☐ substantially short of breath		-11

© 2021 Dinh A et al. *JAMA Network Open.*

☐ terribly short of breath	-13
3a. Do you cough?	
☐ no (skip questions 3b, c and d)	9
☐ only in the morning, when getting up	-6
☐ now and then, all through the day	-6
☐ frequently, all through the day	-12
3b. Do you cough up sputum? (amount of sputum by 24 hrs)	
☐ no	7
☐ less than 2 spoons	-8
☐ more than 2 spoons	-13
☐ half a cup or more	-16
3c. Do you cough up the sputum with ease?	
☐ not bothered by sputum	7
☐ with ease	-9
☐ fairly difficult	-10
☐ very difficult	-10
3d. What is the color of the sputum?	
☐ did not pay attention/no sputum	8
☐ transparent	-8
☐ white	-8
☐ green, yellow or brown	-14
4. I feel fit.	
☐strongly agree	12
☐ agree	4
☐ neither agree or disagree	0
☐ disagree	-6
☐strongly disagree	-11
5. If you were to give a mark on a 1 to 5 scale expressing your general state of health at the moment, which mark would that be?	
☐ excellent	14
☐ good	8
☐ fair	-1
☐ poor	-9
☐ very poor	-15
Raw total (sum) :	_________ (A)
**CAP Score**	**= (A + 99) / 1.69**

Final CAP Score can be comprised between -5.91 (worst) and 101.2 (best).

The CAP score was translated to French for the purpose of the study.

© 2021 Dinh A et al. *JAMA Network Open.*

3. Analysis according to patients' gender

eTable 2. Characteristics of Study Population at Baseline (ie, First Day of β-Lactam) According to Sex

	Male N = 174	Female N = 117	P-value
Failure, n (%)	54 (31.0)	24 (20.5)	0.05*
Age, year, mean (SD)	69.4 (18.3)	70.0 (18.9)	0.76
Comorbidities, n (%)			
Institutionalized	1 (0.6)	8 (6.8)	< 0.01*
Neoplasia	5 (2.9)	1 (0.8)	0.24
Liver failure	3 (1.7)	3 (2.6)	0.62
Heart failure	42 (24.1)	18 (15.4)	0.07
Coronary disease	36 (20.7)	5 (4.3)	< 0.01*
Cerebrovascular disease	12 (6.9)	10 (8.5)	0.60
Renal failure	17 (9.8)	6 (5.1)	0.15
Diabetes mellitus	40 (23.0)	14 (12.0)	0.02*
Chronic lung disease	45 (25.9)	23 (19.7)	0.21
Tobacco use	38 (21.8)	14 (12.0)	0.03*
Clinical signs at Day 0, n (%)			
Dyspnea	90 (51.7)	71 (60.7)	0.13
Cough	141 (81.0)	92 (78.6)	0.62
Sputum production	68 (39.1)	41 (35.0)	0.49
Crackles	138 (79.3)	85 (72.6)	0.19
Confusion	15 (8.6)	10 (8.5)	> 0.99
Pleurisy	3 (1.7)	5 (4.3)	0.19
Delay between first symptom and admission, day, mean (SD)	4.5 (4.5)	5.0 (6.6)	0.47
Patients with 3-day antibiotic treatment, n (%)	84 (48.3)	62 (53.0)	0.06
Antibiotic molecule in the first 3 days, n (%)			
Amoxicillin-clavulanate	112 (64.4)	87 (74.4)	0.07
Third-generation cephalosporins	43 (24.7)	22 (18.8)	0.24
Both molecules	32 (18.4)	14 (12.0)	0.14
Vital signs at Day 0, mean (SD)			
Respiratory rate (/min)	23.8 (6.8)	26.2 (8.2)	0.07
Temperature (°C)	38.9 (0.7)	38.9 (0.6)	0.82
Systolic arterial pressure (mmHg)	135.6 (22.5)	136.9 (26.6)	0.83
Diastolic arterial pressure (mmHg)	73.6 (14.8)	74.4 (14.7)	0.62
Heart rate (beat/min)	101.1 (19.4)	105.8 (19.8)	0.04*
Oxygen saturation (%)	94.6 (3.4)	94.0 (4.4)	0.20

© 2021 Dinh A et al. *JAMA Network Open.*

PSI score at Day 0, mean (SD)	86.8 (30.4)	75.2 (33.0)	< 0.01*
Biologic parameters at Day 0, mean (SD)			
Creatinine clearance (mL/min)	78.1 (25.7)	78.5 (22.0)	0.87
Urea level (mmol/L)	7.9 (4.1)	6.3 (3.3)	< 0.01*
Sodium level (mmol/L)	137.7 (3.8)	136.7 (3.6)	0.02*
Glucose level (mmol/L)	6.8 (2.5)	7.0 (2.8)	0.77
Hematocrit level (%)	39.2 (6.1)	37.6 (4.1)	0.01*
White blood cells count (G/L)	12.0 (5.4)	13.2 (6.0)	0.07
Neutrophils count (G/L)	44.7 (43.0)	11.6 (9.0)	0.41
Platelet count (G/L)	220.3 (108.9)	243.9 (79.9)	< 0.01*
Procalcitonin level (µg/L)	3.4 (8.1)	0.5 (0.7)	< 0.01*
CRP level (mg/L)	135.3 (105.3)	160.6 (147.9)	0.48
Radiography at Day 0, n (%)			
Multi-lobar infection	35 (20.1)	16 (13.7)	0.15
Pleural effusion	17 (9.8)	9 (7.7)	0.54
CAP score at Day 0, mean (SD)	47.8 (37.1)	39.5 (35.9)	0.11
Hospital length of stay, mean (SD)	8.8 (8.0)	8.1 (7.3)	0.45

* Data statistically significant

n: number; SD: standard deviation; CAP: community acquired pneumonia; CRP: C-reactive protein;

PSI: pneumonia severity index

© 2021 Dinh A et al. *JAMA Network Open.*

eReferences

1. El Moussaoui R, Opmeer BC, Bossuyt PMM, Speelman P, de Borgie CAJM, Prins JM. Development and validation of a short questionnaire in community acquired pneumonia. Thorax **2004**; 59:591–5. Available at: http://www.ncbi.nlm.nih.gov/pubmed/15223867. Accessed 4 October 2019.

© 2021 Dinh A et al. *JAMA Network Open.*
