# Supplementary material for: Factors Associated With Treatment Failure in Moderately Severe Community-Acquired Pneumonia: A Secondary Analysis of a Randomized Clinical Trial
Source: JAMA Netw Open. 2021 Oct 15;4(10):e2129566. doi: 10.1001/jamanetworkopen.2021.29566 (PMC8520128; doi:10.1001/jamanetworkopen.2021.29566)
Supplement: Supplement 2. — Nonauthor Collaborators. Pneumonia Short Treatment Study Group Members [file jamanetwopen-e2129566-s002.pdf]

Supplemental Online Content: Nonauthor Collaborators

*Indicates required information. Only first name, last name, and suffix will appear in PubMed.

*Group Name(s): Pneumonia Short Treatment (PTC) Study Group							
***First Name and Middle Initial(s)**	***Last Name**	***Suffix (eg, Jr, III)**	Academic Degrees	Institution	Location (city, state/province, country)	Role or Contribution, eg, chair, principal investigator	Group (if more than 1 Group listed in the byline) and/or Subgroup (eg, Steering Committee)
Julie	ATTAL-BEHAR		MD	Ambroise Paré University Hospital, AP-HP Paris Saclay University	Boulogne-Billancourt, France		
Sébastien	BEAUNE		MD	Ambroise Paré University Hospital, AP-HP Paris Saclay University	Boulogne-Billancourt, France		
Thierry	CHINET		MD, PhD	Ambroise Paré University Hospital, AP-HP Paris Saclay University	Boulogne-Billancourt, France		
Tristan	CUDENNEC		MD	Ambroise Paré University Hospital, AP-HP Paris Saclay University	Boulogne-Billancourt, France		
Marine	DE LAROCHE		MD	Ambroise Paré University Hospital, AP-HP Paris Saclay University	Boulogne-Billancourt, France		
Albane	DE THEZY		MD	Ambroise Paré University Hospital, AP-HP Paris Saclay University	Boulogne-Billancourt, France		
Jennifer	DUMOULIN		MD	Ambroise Paré University Hospital, AP-HP Paris Saclay University	Boulogne-Billancourt, France		
Caroline	DUPONT		MD	Ambroise Paré University Hospital, AP-HP Paris Saclay University	Boulogne-Billancourt, France		
Elise	FERCOT		MD	Ambroise Paré University Hospital, AP-HP Paris Saclay University	Boulogne-Billancourt, France		

*Indicates required information. Only first name, last name, and suffix will appear in PubMed.

*First Name and Middle Initial(s)	*Last Name	*Suffix (eg, Jr, III)	Academic Degrees	Institution	Location (city, state/province, country)	Role or Contribution, eg, chair, principal investigator	Group (if more than 1 Group listed in the byline) and/or Subgroup (eg, Steering Committee)
Violaine	GIRAUT		MD	Ambroise Paré University Hospital, AP-HP Paris Saclay University	Boulogne-Billancourt, France		
Ségolène	GREFFE		MD	Ambroise Paré University Hospital, AP-HP Paris Saclay University	Boulogne-Billancourt, France		
Julie	GRENET		MD	Ambroise Paré University Hospital, AP-HP Paris Saclay University	Boulogne-Billancourt, France		
Caroline	GUYOT		MD	Ambroise Paré University Hospital, AP-HP Paris Saclay University	Boulogne-Billancourt, France		
Jean-Emmanuel	KAHN		MD, PhD	Ambroise Paré University Hospital, AP-HP Paris Saclay University	Boulogne-Billancourt, France		
Sylvie	LABRUNE		MD	Ambroise Paré University Hospital, AP-HP Paris Saclay University	Boulogne-Billancourt, France		
Marie	LACHATRE		MD	Ambroise Paré University Hospital, AP-HP Paris Saclay University	Boulogne-Billancourt, France		
Sophie	MOULIAS		MD	Ambroise Paré University Hospital, AP-HP Paris Saclay University	Boulogne-Billancourt, France		
Charlotte	NALINE		MD	Ambroise Paré University Hospital, AP-HP Paris Saclay University	Boulogne-Billancourt, France		
Marion	PEPIN		MD	Ambroise Paré University Hospital, AP-HP Paris Saclay University	Boulogne-Billancourt, France		

*Indicates required information. Only first name, last name, and suffix will appear in PubMed.

***First Name and Middle Initial(s)**	***Last Name**	***Suffix (eg, Jr, III)**	Academic Degrees	Institution	Location (city, state/province, country)	Role or Contribution, eg, chair, principal investigator	Group (if more than 1 Group listed in the byline) and/or Subgroup (eg, Steering Committee)
Elisabeth	ROUVEIX		MD, PhD	Ambroise Paré University Hospital, AP-HP Paris Saclay University	Boulogne-Billancourt, France		
Marine	SAHUT-D'IZARN		MD	Ambroise Paré University Hospital, AP-HP Paris Saclay University	Boulogne-Billancourt, France		
Abel	SEFSSAFI		MD	Ambroise Paré University Hospital, AP-HP Paris Saclay University	Boulogne-Billancourt, France		
Laurent	TEILLET		MD, PhD	Ambroise Paré University Hospital, AP-HP Paris Saclay University	Boulogne-Billancourt, France		
Jean-Pierre	BRU		MD	Annecy Hospital	Annecy, France		
Jacques	GAILLAT		MD	Annecy Hospital	Annecy, France		
Vincent	GAUTIER		MD	Annecy Hospital	Annecy, France		
Cécile	JANSSEN		MD	Annecy Hospital	Annecy, France		
Leonardo	PAGANI		MD	Annecy Hospital	Annecy, France		
Virginie	VITRAT		MD	Annecy Hospital	Annecy, France		
Malika	ABDERRAHMANE		MD	Argenteuil Hospital	Argenteuil, France		
Juliette	CAMUSET		MD	Argenteuil Hospital	Argenteuil, France		
Catherine	LEGALL		MD	Argenteuil Hospital	Argenteuil, France		
Pascale	LONGUET-FLANDRES		MD	Argenteuil Hospital	Argenteuil, France		
Anne-Marie	MENN		MD	Argenteuil Hospital	Argenteuil, France		
Victoire	DE LASTOURS		MD, PhD	Beaujon University Hospital, AP-HP	Clichy, France		
Marie	LECRONIER		MD	Beaujon University Hospital, AP-HP	Clichy, France		
Gwenolée	PREVOST		MD	Beaujon University Hospital, AP-HP	Clichy, France		

*Indicates required information. Only first name, last name, and suffix will appear in PubMed.

*First Name and Middle Initial(s)	*Last Name	*Suffix (eg, Jr, III)	Academic Degrees	Institution	Location (city, state/province, country)	Role or Contribution, eg, chair, principal investigator	Group (if more than 1 Group listed in the byline) and/or Subgroup (eg, Steering Committee)
Charles	BURDET		MD	Bicêtre University Hospital, AP-HP Paris Saclay University	Le Kremlin-Bicêtre, France		
Ouda	DERRADJI		MD	Bicêtre University Hospital, AP-HP Paris Saclay University	Le Kremlin-Bicêtre, France		
Lelia	ESCAUT		MD	Bicêtre University Hospital, AP-HP Paris Saclay University	Le Kremlin-Bicêtre, France		
Etienne	HINGLAIS		MD	Bicêtre University Hospital, AP-HP Paris Saclay University	Le Kremlin-Bicêtre, France		
Philippe	LEBRAS		MD	Bicêtre University Hospital, AP-HP Paris Saclay University	Le Kremlin-Bicêtre, France		
Edouard	LEFEVRE		MD	Bicêtre University Hospital, AP-HP Paris Saclay University	Le Kremlin-Bicêtre, France		
Mathilde	NOAILLON		MD	Bicêtre University Hospital, AP-HP Paris Saclay University	Le Kremlin-Bicêtre, France		
Pauline	RABIER		MD	Bicêtre University Hospital, AP-HP Paris Saclay University	Le Kremlin-Bicêtre, France		
Maurice	RAPHAEL		MD	Bicêtre University Hospital, AP-HP Paris Saclay University	Le Kremlin-Bicêtre, France		
Elina	TEICHER		MD	Bicêtre University Hospital, AP-HP Paris Saclay University	Le Kremlin-Bicêtre, France		
Christiane	VERNY		MD	Bicêtre University Hospital, AP-HP Paris Saclay University	Le Kremlin-Bicêtre, France		
Daniel	VITTECOQ		MD, PhD	Bicêtre University Hospital, AP-HP Paris Saclay University	Le Kremlin-Bicêtre, France		
Benjamin	WYPLOSZ		MD	Bicêtre University Hospital, AP-HP Paris Saclay University	Le Kremlin-Bicêtre, France		
Michèle	BEN HAYOUN		MD	Bichât University Hospital, AP-HP	Paris, France		
Françoise	BRUN-VEZINET		MD	Bichât University Hospital, AP-HP	Paris, France		

*Indicates required information. Only first name, last name, and suffix will appear in PubMed.

***First Name and Middle Initial(s)**	***Last Name**	***Suffix (eg, Jr, III)**	Academic Degrees	Institution	Location (city, state/province, country)	Role or Contribution, eg, chair, principal investigator	Group (if more than 1 Group listed in the byline) and/or Subgroup (eg, Steering Committee)
Enrique	CASALINO		MD	Bichât University Hospital, AP-HP	Paris, France		
Christophe	CHOQUET		MD	Bichât University Hospital, AP-HP	Paris, France		
Marie-Christine	DOMBRET		MD	Bichât University Hospital, AP-HP	Paris, France		
Xavier	DUVAL		MD, PhD	Bichât University Hospital, AP-HP	Paris, France		
Nadhira	HOUHOU		MD	Bichât University Hospital, AP-HP	Paris, France		
Véronique	JOLY		MD	Bichât University Hospital, AP-HP	Paris, France		
Xavier	LESCURE		MD, PhD	Bichât University Hospital, AP-HP	Paris, France		
Manuela	POGLIAGHI		MD	Bichât University Hospital, AP-HP	Paris, France		
Christophe	RIOUX		MD	Bichât University Hospital, AP-HP	Paris, France		
Yazdan	YAZDANPANAH		MD, PhD	Bichât University Hospital, AP-HP	Paris, France		
Elsa	BARROS		MD	Créteil University Hospital	Créteil, France		
Belinda	BEGGA		MD	Créteil University Hospital	Créteil, France		
Sébastien	BOUKOBZA		MD	Créteil University Hospital	Créteil, France		
Houria	BOUREDJI		MD	Créteil University Hospital	Créteil, France		
Imad	CHOUAHI		MD	Créteil University Hospital	Créteil, France		
Isabelle	DELACROIX		MD	Créteil University Hospital	Créteil, France		
Antoine	FROISSART		MD	Créteil University Hospital	Créteil, France		
Valérie	GARRAIT		MD	Créteil University Hospital	Créteil, France		
Elsa	NGWEM		MD	Créteil University Hospital	Créteil, France		
Catherine	PHLIPPOTEAU		MD	Créteil University Hospital	Créteil, France		
Sepehr	SALEHABADI		MD	Créteil University Hospital	Créteil, France		

*Indicates required information. Only first name, last name, and suffix will appear in PubMed.

***First Name and Middle Initial(s)**	***Last Name**	***Suffix (eg, Jr, III)**	Academic Degrees	Institution	Location (city, state/province, country)	Role or Contribution, eg, chair, principal investigator	Group (if more than 1 Group listed in the byline) and/or Subgroup (eg, Steering Committee)
Cécile	TOPER		MD	Créteil University Hospital	Créteil, France		
Florent	VINAS		MD	Créteil University Hospital	Créteil, France		
Marie	AMSILLI		MD	Grenoble Hospital	Grenoble, France		
Olivier	EPAULARD		MD	Grenoble Hospital	Grenoble, France		
Patricia	PAVESE		MD	Grenoble Hospital	Grenoble, France		
Isabelle	PIERRE		MD	Grenoble Hospital	Grenoble, France		
Jérôme	AULAGNIER		MD	Foch Hospital	Suresnes, France		
Julie	CELERIER		MD	Foch Hospital	Suresnes, France		
Roxana	COJOCARIU		MD	Foch Hospital	Suresnes, France		
Emmanuel	MATHIEU		MD	Foch Hospital	Suresnes, France		
Charlotte	RACHLINE		MD	Foch Hospital	Suresnes, France		
Yoland	SCHOINDRE		MD	Foch Hospital	Suresnes, France		
Thomas	SENE		MD	Foch Hospital	Suresnes, France		
Christelle	THIERRY		MD	Foch Hospital	Suresnes, France		
Caroline	APARICIO		MD	Lariboisière University Hospital, AP-HP	Paris, France		
Véronique	DELCEY		MD	Lariboisière University Hospital, AP-HP	Paris, France		
Amanda	LOPES		MD	Lariboisière University Hospital, AP-HP	Paris, France		
Marjolaine	MORGAND		MD	Lariboisière University Hospital, AP-HP	Paris, France		
Pierre	SELLIER		MD	Lariboisière University Hospital, AP-HP	Paris, France		
Guy	SIMONEAU		MD	Lariboisière University Hospital, AP-HP	Paris, France		
Catherine	CHAKVETADZE		MD	Melun Hospital	Melun, France		
Sylvain	DIAMANTIS		MD	Melun Hospital	Melun, France		
Arnaud	GAUTHIER		MD	Melun Hospital	Melun, France		
Kaoutar	JIDAR		MD	Melun Hospital	Melun, France		

*Indicates required information. Only first name, last name, and suffix will appear in PubMed.

*First Name and Middle Initial(s)	*Last Name	*Suffix (eg, Jr, III)	Academic Degrees	Institution	Location (city, state/province, country)	Role or Contribution, eg, chair, principal investigator	Group (if more than 1 Group listed in the byline) and/or Subgroup (eg, Steering Committee)
Béatrice	JOURDAIN		MD	Melun Hospital	Melun, France		
Jean-Francois	BOITIAUX		MD	Pontoise Hospital	Pontoise, France		
Patrick	DESCHAMPS		MD	Pontoise Hospital	Pontoise, France		
Edouard	DEVAUD		MD	Pontoise Hospital	Pontoise, France		
Bruno	PHILIPPE		MD	Pontoise Hospital	Pontoise, France		
Ruxandra-Oana	CALIN		MD	Raymond Poincaré Hospital, AP-HP Paris Saclay University	Garches, France		
Tomasz	CHROBOCZEK		MD	Raymond Poincaré Hospital, AP-HP Paris Saclay University	Garches, France		
Benjamin	DAVIDO		MD	Raymond Poincaré Hospital, AP-HP Paris Saclay University	Garches, France		
Laurène	DECONINCK		MD	Raymond Poincaré Hospital, AP-HP Paris Saclay University	Garches, France		
Pierre	DE TRUCHIS		MD	Raymond Poincaré Hospital, AP-HP Paris Saclay University	Garches, France		
Aurore	LAGRANGE		MD	Raymond Poincaré Hospital, AP-HP Paris Saclay University	Garches, France		
Sabrina	MAKHLOUFI		MD	Raymond Poincaré Hospital, AP-HP Paris Saclay University	Garches, France		
Morgan	MATT		MD	Raymond Poincaré Hospital, AP-HP Paris Saclay University	Garches, France		
Guillaume	MELLON		MD	Raymond Poincaré Hospital, AP-HP Paris Saclay University	Garches, France		
Olivia	SENARD		MD	Raymond Poincaré Hospital, AP-HP Paris Saclay University	Garches, France		
Daniel	BENHAMOU		MD	Rouen Hospital	Rouen, France		
Claire	CHAPUZET		MD	Rouen Hospital	Rouen, France		
Laure	CHAUFFREY		MD	Rouen Hospital	Rouen, France		
Manuel	ETIENNE		MD	Rouen Hospital	Rouen, France		
Luc-Marie	JOLY		MD	Rouen Hospital	Rouen, France		
Bérengère	OBSTOY		MD	Rouen Hospital	Rouen, France		

Supplemental Online Content: Nonauthor Collaborators

*Indicates required information. Only first name, last name, and suffix will appear in PubMed.

***First Name and Middle Initial(s)**	***Last Name**	***Suffix (eg, Jr, III)**	Academic Degrees	Institution	Location (city, state/province, country)	Role or Contribution, eg, chair, principal investigator	Group (if more than 1 Group listed in the byline) and/or Subgroup (eg, Steering Committee)
Mathieu	SALAUN		MD	Rouen Hospital	Rouen, France		
Luc	THIBERVILLE		MD	Rouen Hospital	Rouen, France		
Julie	TILLON		MD	Rouen Hospital	Rouen, France		
Diane	BOLLENS		MD	Saint-Antoine University Hospital, AP-HP	Paris, France		
Julie	BOTTERO		MD	Saint-Antoine University Hospital, AP-HP	Paris, France		
Pauline	CAMPA		MD	Saint-Antoine University Hospital, AP-HP	Paris, France		
Gäelle	COSQUERIC		MD	Saint-Antoine University Hospital, AP-HP	Paris, France		
Bénédicte	LEFEBVRE		MD	Saint-Antoine University Hospital, AP-HP	Paris, France		
Zineb	OUAZENE		MD	Saint-Antoine University Hospital, AP-HP	Paris, France		
Jérôme	PACANOWSKI		MD	Saint-Antoine University Hospital, AP-HP	Paris, France		
Dominique	PATERON		MD	Saint-Antoine University Hospital, AP-HP	Paris, France		
Nadia	VALIN		MD	Saint-Antoine University Hospital, AP-HP	Paris, France		
Caroline	COMPAIN		MD	Saint-Denis Hospital, AP-HP	Saint-Denis, France		
Hugues	CORDEL		MD	Saint-Denis Hospital, AP-HP	Saint-Denis, France		
Benoit	DOUMENC		MD	Saint-Denis Hospital, AP-HP	Saint-Denis, France		
Elena	FOIS		MD	Saint-Denis Hospital, AP-HP	Saint-Denis, France		
Nicolas	GAMBIER		MD	Saint-Denis Hospital, AP-HP	Saint-Denis, France		
Marie-Aude	KHUONG		MD	Saint-Denis Hospital, AP-HP	Saint-Denis, France		
Elisa	PASQUALONI		MD	Saint-Denis Hospital, AP-HP	Saint-Denis, France		
Marie	POUPARD		MD	Saint-Denis Hospital, AP-HP	Saint-Denis, France		
